# Peer Pressure, Psychological Distress and the Urge to Smoke

**DOI:** 10.3390/ijerph6061799

**Published:** 2009-06-10

**Authors:** Yi-Wen Tsai, Yu-Wen Wen, Chia-Rung Tsai, Tzu-I Tsai

**Affiliations:** 1 Institute of Population Health Sciences / National Health Research Institutes, No.35, Keyan Road, Zhunan Town, Miaoli County 350, Taiwan; E-Mails: ivytsai@nhri.org.tw (Y.W.T.); ywwen@nhri.org.tw (Y.W.W.); ttsaii@nhri.org.tw (C.R.T.); 2 School of Nursing / National Yang Ming University / No.155, Sec2, Linong St., Beitou District, Taipei City 112, Taiwan

**Keywords:** cue response, psychological motive, social motive, smoking behavior

## Abstract

**Background:**

Psychology and addiction research have found that cigarette smokers react with subjective and automatic responses to stimuli associated with smoking. This study examines the association between the number of cigarettes smokers consume per month and their response to cues derived from peer and psychological distress.

**Methods:**

We studied 1,220 adult past and current smokers drawn from a national face-to-face interview survey administered in 2004. We defined two types of cues possibly triggering a smoker to have a cigarette: peer cues and psychological cues. We used ordinary least square linear regressions to analyze smoking amount and response to peer and psychological distress cues.

**Results:**

We found a positive association between amount smoked and cue response: peer cues (1.06, 95%CI: 0.74–1.38) and psychological cues (0.44, 95%CI = 0.17–0.70). Response to psychological cues was lower among male smokers (−1.62, 95%CI = −2.26–−0.98), but response to psychological cues were higher among those who had senior high school level educations (0.96, 95%CI = 0.40–1.53) and who began smoking as a response to their moods (1.25, 95%CI = 0.68–1.82).

**Conclusions:**

These results suggest that both peer cues and psychological cues increase the possibility of contingent smoking, and should, therefore, be addressed by anti-smoking policies and anti-smoking programs. More specifically, special attention can be paid to help smokers avoid or counter social pressure to smoke and to help smokers resist the use of cigarettes to relieve distress.

## 1. Introduction

Tobacco smoking is the world’s largest preventable cause of disease morbidity and mortality, and tobacco control policies play a very important role in reducing the economic cost of tobacco to society and the individual. Although prevention is an important aim of tobacco control, so is cessation. Cessation without assistance is very difficult due to the highly addictive nature of cigarettes and unpleasantness of withdrawing from nicotine [1–4]. Current treatment guidelines recommend healthcare providers advise every smoker to quit and provide treatment for his or her dependence [5,6].

Although there is an increase in effective and economical modes of treatment for tobacco dependence, including behavioral counseling and pharmacotherapy [5,7–9], such treatment remains underused [10] and the relapse rates are very high [11–13]. Relapse after the 12th month of cession range from 10% to 28.4% [11,13], reducing the apparent effectiveness of various treatments for this addiction [11].

What keeps a smoker from successfully quitting cigarettes is an important question, not only for clinicians but also for policymakers and health economists. Clinically, most treatment protocols adhere to the well-known 5A’s model [5], which is to ask (screen for smoking), advise (provide a quit message), assess (evaluate readiness to quit), assist (provide treatment) and arrange (track cessation progress). Most providers who try to help their patients overcome their addiction to cigarettes prescribe nicotine patches, bupropion, or nicotine replacement therapy [14]. However, long-term relapse rates show that it will take more than pharmaceuticals to overcome the addiction to nicotine. Nicotine has been found to act in the midbrain, where it creates impulses to smoke in response to stimuli [15]. In addition, some studies applied the Pavlovian’s conditioning theory or social learning theories to highlight the role of environmental events or affective/physiological cues that may evoke conditioned reactions to substance use [16–20].

In tobacco and addiction research, craving, defined as a subjective automatic response to related stimuli, is thought to contribute to the continuance of smoking and relapse [21–25]. The concept of cigarette craving is often measured by using cue reactivity methods as cue reactivity has proven useful in studying addictive behaviors and is used as a treatment strategy for relapse prevention [18,26–28]. Previous evidence suggests that affective experiences (i.e. positive or negative emotions, feeling stressed) and social situations (i.e. being in the company of others, with smokers present, while eating and/or drinking) can elicit urges to smoke [29–32]. However, most studies have been done based on the laboratory experiments by using “visible” photographs, role-play situation or imagery scripts to investigate smokers’ mood, physiological changes, or self-reported craving [18,31,33,34]. Although laboratory-based approach offers greater potential for experimental manipulation of tobacco-specific cues, this approach is limited to whether there is a meaningful association between the cue-elicited craving and real smoking behavior. Smoking behavior is considered to be a set of complex responses to smoking cues which interact with the given social-environmental context. Some studies have noted that the patterning of smoking cues related to reactivity and the responses to cues vary according to smoking history, gender, and social situations [32,34,35]. The current literature has mainly documented the empirical findings in Western societies, and there is little information on cue responses among non-Western communities. The present study used a population-based sample from Taiwan to investigate the relationship between cue responses and the amount of cigarettes smoked among current and former smokers.

## 2. Materials and Methods

### 2.1. Data Collection

This study draws from data obtained in a three-stage random-sampled face-to-face interview survey on cigarette consumption administered in 107 townships and two metropolitan areas in Taiwan during 2004. We surveyed 3,874 non-institutionalized residents (1,908 men and 1,966 women) aged 18 or above. The surveys were reviewed and approved by the Institutional Review Board of National Health Research Institutes. Interviewees were asked to sign an informed consent detailing the purpose of the study, benefits and risks associated with participation and assuring confidentiality.

Of the 3,874 adult subjects surveyed in 2004, 1,104 (57.86%) of the 1,908 men and 116 (5.90%) of the 1,966 women were current smokers or past-smokers. Nine hundred and twenty-seven of the smokers (75.98%) were current smokers. The 2004 prevalence rates were similar to those found in the 2001 National Health Interview Survey (46.5% in men and 4.2% in women) [36].

### 2.2. Measures

***Cue Response (CR).*** Cue responses consist of six items. The factor analysis confirms two factors (peer cues and psychological cues) which account for approximately 68 percent of variance. Psychometric measurement indicates the overall internal consistency is 0.79. Peer cues (CR_peer) measured how sensitive a smoker was to people smoking around him or her. The higher the value for CR_peer, the more likely it would be that the respondent would smoke when he or she saw someone smoking. Psychological cues (CR_psych) measured how likely it would be for a respondent to smoke when he or she was in a bad mood or stressed. The higher the CR psych value, the stronger the desire to smoker when challenged. The measures of these two CRs were defined based on the answers to the questions regarding the desire to smoke in different circumstances: 0 meaning not at all, 1 little, 2 moderate, 3 very much, and 4 extreme. CR_peer was calculated by summing up the answers for three circumstances: (1) being among smokers in a party or wedding banquet, (2) seeing someone else smoke, and (3) being around friends who are smoking. CR_psych was calculated by summing up the answers for three circumstances: (1) feeling nervous and anxious, (2) feeling angry, and (3) feeling frustrated. Each cue was scored from 0 to 12.

***Smoking behavior and independent variables.*** The dependent variable, current amount smoked (CAS) was measured based on how many packs of cigarettes a smoker consumed per month. The current amount smoked for past smokers was defined as zero. Two variables were defined to represent the reasons people started smoking in the beginning: SOCIAL_MOTIVE and MOOD_MOTIVE. On the survey, smokers were asked to select from a list of eight possible reasons for beginning to smoke, including “to satisfy initial curiosity”, “to stimulate and maintain vitality”, “to enhance positive sensations”, “to control body weight”, or “to alleviate stress” on in response to “a tobacco advertisement” or “social activity”. They could choose more than one reason. They were assigned a SOCIAL_MOTIVE score of 1 if they indicated that they started for reasons of social interaction, and zero if not. They were assigned a MOOD_MOTIVE of 1 if they started as a means of coping with stress and zero if not. Dummy variables representing exposure to cigarette advertising, exposure to family smoke at home, and exposure to second-hand smoke in the workplace were assigned a value of 1 if participants indicated they has been exposed this in one of these ways and zero if not. Sociodemographic factors included gender, age, educational level, and employment status.

### 2.3. Statistical Methods

Linear regressions were used to analyze the amount smoked (CAS), cue-response to peer (CR_peer) and cue-response to psychological effect (CR_psych). First, ordinary least square (OLS) estimation was used to estimate regressions of CAS, CR_peer, and CR_psych with each of these variables, which were also included as independent variables in the estimations of the others. All models and tests included the total ever-smokers as well on male smokers alone. We also used the Hausman test [37] to check the endogeneity when some independent variables were correlated with the error term. The endogeneity problem may lead the biased estimate in OLS estimation. All statistical operations were performed using STATA version 7.

## 3. Results

Table 1 shows the descriptive data of the 1,220 smokers: 927 current smokers (836 males, 91 females) and 293 former smokers (268 males, 25 females). Two hundred and sixty-three (21.56%) reported that they first started smoking for social reasons (SOCIAL_MOTIVE) and 143 (11.72%) for stress relief (MOOD_MOTIVE). Most smokers, both former and current, reported being reactive to both peer and psychological cues (96.97% and 91.48%, respectively). Those who tended to smoke most were 35–54 years old (26.30 to 28.61 packs per month), male (26.26, Std = 15.74), and had junior high school levels of education (27.09, Std = 15.78) or below (27.34, Std = 17.45). Female smokers were different from male smokers in a few ways. Most obviously, only 9.48% of the women reported a social motive for first time smoking, in contrast to 22.83% of male smokers, but a higher percentage of women than men reported that they started in an effort to manage their moods (27.89% vs. 10.05%, respectively).

In Table 2, the average CR_peer scores and CR_psych scores were 6.25 (Std = 2.64) and 5.75 (Std = 3.34), respectively. Of the 1,220 smokers, 1,183 (96.97%) reported being reactive to peer cues (CR_peer > 0), mostly men (97.19% vs. 94.83%). The men also reported a higher response to peer cues than females (CR_peer 6.35, Std = 2.62 vs. 5.31, Std = 2.69. respectively). Another group difference in CR_peer was whether the smokers originally started smoking to manage moods. While a higher proportion of those who originally began smoking to manage their moods reported being responsive to positive CR_peer (97.90% vs. 96.84%), they had a lower average (mean = 5.92 vs. 6.29) than those who originally were not mood-motivated to start.

While most smokers (1,116, 91.48%) reported being reactive to psychological cues (CR_psych > 0), there were significant differences in CR_psych scores when analyzed by age, gender and education. Smokers over 55 years old were less likely to report being reactive to psychological cues (97.54% vs. 87.54%) and also had lower mean scores in this measure than younger groups (mean = 4.59 vs. 6.99). A greater proportion of female smokers reported being reactive to psychological cues than male smokers (94.83% vs. 91.12%), and these women also reported a higher mean response (7.48 vs. 5.57). A greater proportion of smokers (94.39%) with senior high school educations reported being reactive to psychological cues, reporting a higher mean CR_psych score (6.49, Std = 3.28) than smokers at other education levels.

Table 3a and Table 3b show the results of single-equation on amount smoked, CR_peer and CR_psych for total smokers and male smokers. The Hausman tests showed insignificant endogeneity in the regression on amount smoked, CR_peer and CR_psych. As can be seen in Table 3a for all smokers, there was a positive association between amount smoked and increased CR_peer values (0.05, 95%CI = 0.04–0.06) and CR_psych values (0.05, 95%CI = 0.03–0.06). There was also a negative association between CR_peer and employment status (−0.52, 95%CI = −0.97–−0.07), and a positive effect between CR_peer and exposure to secondhand smoke in the workplace (0.49, 95%CI = 0.12–0.85). The degree of response to psychological cues was lower for male smokers than female smokers (−1.62, 95%CI = −2.26–−0.98). Smokers who started smoking to cope with mood (MOOD_MOTIVE) tended to report higher reactivity to psychological cues (1.25, 95%CI = 0.68–1.82). Smokers aged 35–44, 45–54 and 55 years or above tended to report lower response to psychological cues than smokers aged 18–24 years (−0.98, 95%CI = −1.66–−0.30, −1.02, 95%CI = −1.74–−0.30 and −1.79, 95%CI = −2.57–−1.01). Smokers with senior high school levels (0.96, 95%CI = 0.40–1.53) reported higher response to psychological cues than those with elementary school or no education. Current smokers had higher smoking amount than former smokers (24.93, 95%CI = 23.06–26.80). Current smoker reported lower response to peer and psychological cues than former smokers (−1.37, 95%CI = −1.84–−0.91 and −1.19, 95%CI = −1.75–−0.63). The estimates for total smokers were very similar to those for male smokers (Table 3b).

## 4. Discussion and Conclusions

This study used a national sample to investigate the influence of external peer cues and internal psychological cues that might cause a smoker to want to have a cigarette in Taiwan. The findings suggest that response to peer cues plays a very important role in the smoking behavior of men in Taiwan and psychological cues is more important for women who are more likely to use smoking as a means of coping with psychological distress.

This study extends the existing research on cue-reactivity in addiction [27,28,33,38,39]. Most previous studies have investigated the influence of photographs or imagery scripts as cues to test reactivity in subjects who self-reported cravings or mood states or in subjects whose physiological changes such as heart rate or skin conductance were compared. This study measured responses to other variables, such as seeing others smoke and internally emerging psychological stimuli.

We extend and strengthen the current public health and economic research on tobacco control policies [12,39,40]. While most tobacco control polices have been based on price elasticity, regulation, health education aimed at imparting health information and enhancing knowledge regarding the hazards of smoking over the past 40 years, tobacco marketers have paid increasing attention to smokers’ psychological and attitudinal profiles, physical environment, and activities. They have changed from marketing product characteristics to a general public to targeting their markets physchographically. This study provides public health with greater insight into smoking behavior by looking into both environmental and affective cue-triggered smoking behavior and may help current tobacco control policies become more multi-dimensional.

The findings of this study show that response to peer cues plays a very important role in the behavior of smokers. The stronger the urge induced by peer cues, the greater the amount smoked. Most tobacco control literature emphasizes the importance of peer effect with regard to its influence on teenagers and much effort has devoted to educating this high-risk group. These findings indicate that, in addition to nicotine patches and regular health education, more should be done to help the smoker learn to avoid or counter such cues triggering smoking. Current cue-exposure treatments have shown promise in treating some addictive disorders [38,39], and may be useful for relapse prevention in the smoking cessation treatment [41,42]. In terms of the public health prevention aspect, we propose an integration of cue exposure methods to break the cue-elicited association through eliminating environmental cues and regulating cigarette advertisements in future anti-tobacco policies.

Another significant finding of this study regards the influence exerted by psychological cues on amount smoked. The influence of psychological cues can be traced back to the first time a person started smoking. The urge to smoke for some people may be born from an internal need to calm themselves down, not from a need to fit in socially. This study found women who smoked tended to be more reactive to psychological triggers than men, suggesting that women might be more likely to use smoking to temper emotions as a means of coping with stress, anxiety and depression than men. Current smoker tended to be more reactive to peer and psychological cues than former smokers. The findings of this study indicate that more needs to be done to provide anti-tobacco programs and campaigns aimed at detaching the smoking of cigarettes from the soothing of emotions, an approach conspicuously missing in tobacco control programs policy.

This study is subject to the limitations associated with use of survey data: subjective information and recall bias. With self-reported cue response to particular circumstances, there may exist a certain bias derived by subjectivity. Also, because the rate of smoking among women is very low in Taiwan, the study sample of female smokers was very small, limiting the generalization of our results to the general female population. Nevertheless, this population-based study contributes to our understanding of cigarette addiction, especially by reporting that social and psychological cues may induce smoking over diverse sociodemographic backgrounds. Future anti-tobacco prevention and control campaigns should be tailored to fit the psychological and attitudinal profiles of potential and current smokers. In Taiwan, more health education should be given to male smokers to help them learn to resist smoking when challenged by social stress, and more social support or consultation should be provided to women to help them resist smoking caused from psychological stress.

## Figures and Tables

**Table 1.** Descriptive statistics of the study sample of 1,220 current and past smokers.

	**Total (n = 1,220)**		**Male smokers (n = 1,104)**		**Female Smokers (n = 116)**		**Monthly Amount Smoked (packs)**							
							**Total smokers**		**Current smokers**		**Male smokers**		**Current male smokers**	
	N	%	N	%	N	%	Mean	Std	Mean	Std	Mean	Std	Mean	Std
**Total**	1,220	100.00	1,104	100.00	116	100.00	19.19	17.39			19.86	17.74		
**Current smokers**	927	75.98	836	75.72	91	78.45			25.28	15.63			26.26	15.74
**Age (years)**														
18–24	122	10.00	99	8.97	23	19.83	17.29	11.43	19.35	10.30	18.47	11.60	20.09	10.66
25–34	204	16.72	174	15.76	30	25.86	22.38	15.22	24.82	14.01	23.73	15.30	25.66	14.26
35–44	290	23.77	259	23.46	31	26.72	21.75	17.97	26.30	16.45	23.14	18.18	27.90	16.29
45–54	245	20.08	227	20.56	18	15.52	21.78	19.60	28.61	17.58	22.25	20.00	29.45	17.80
55+	321	26.31	309	27.99	12	10.34	14.02	17.23	24.79	16.04	14.12	17.27	25.28	15.86
Missing	38		36		2									
**Gender**														
Woman	116	9.51					12.83	11.94	16.36	11.12				
Man	1,104	90.49					19.86	17.74	26.26	15.74				
**Education**														
Elementary school or no education	305	25.00	280	25.36	25	21.55	19.58	19.24	27.34	17.45	19.90	19.63	28.37	17.56
Junior high school	237	19.43	214	19.38	23	19.83	21.69	17.80	27.09	15.78	22.44	18.24	28.28	15.93
Senior high school	553	45.33	492	44.57	61	52.59	19.55	16.37	24.53	14.62	20.55	16.62	25.55	14.68
Undergraduate or graduate school	122	10.00	115	10.42	7	6.03	11.65	14.04	18.94	13.49	11.95	14.28	19.36	13.66
Missing	3		3											
**Employment Status**														
No	387	31.72	329	29.80	58	50.00	15.57	17.96	24.47	17.00	16.20	18.73	26.36	17.40
Yes	831	68.11	773	70.02	58	50.00	20.88	16.87	25.57	15.11	21.42	17.08	26.22	15.21
Missing	2		2											
**Social motive for the first-time smoking**														
No	957	78.44	852	77.17	105	90.52	19.30	16.83	24.95	15.01	20.05	17.20	26.07	15.09
Yes	263	21.56	252	22.83	11	9.48	18.77	19.31	26.62	17.87	19.20	19.47	26.96	17.96
**Mood motive for the first-time smoking**														
No	1077	88.28	993	89.95	84	72.41	19.26	17.69	25.68	15.89	19.75	18.01	26.44	16.03
Yes	143	11.72	111	10.05	32	27.59	18.63	14.94	22.57	13.45	20.80	15.18	24.83	13.20
**Cue response**														
CR_Peer>0	1183	96.97	1073	97.19	110	94.83	19.58	17.42	25.61	15.57	20.26	17.75	26.56	15.67
CR_Psych>0	1116	91.48	1006	91.12	110	94.83	19.89	17.40	25.80	15.49	20.66	17.72	26.88	15.54
**Exposure to cigarette advertisement**														
No	566	46.39	532	48.19	34	29.31	19.61	18.03	26.73	15.90	19.92	18.26	27.31	15.97
Yes	654	53.61	572	51.81	82	70.69	18.82	16.82	24.11	15.32	19.79	17.26	25.35	15.51
**Exposure to second hand smoke in the workplace**														
No	668	54.75	620	56.16	48	41.38	16.65	17.62	24.16	16.39	17.13	17.87	24.91	16.44
Yes	515	42.21	483	43.75	32	27.59	23.04	16.69	27.12	14.73	23.40	16.94	27.67	14.87
Missing	37		1		36	31.03								
**Exposure to second hand smoke at home**														
No	732	60.00	697	63.13	35	30.17	17.80	17.47	25.40	15.57	18.14	17.62	25.77	15.64
Yes	488	40.00	407	36.87	81	69.83	21.26	17.07	25.13	15.72	22.78	17.57	26.96	15.89

Std: standard deviation

**Table 2.** Descriptive statistics on cue response.

	**Cue Response on Peer (CR_Peer)**								**Cue Response on Psychological Stimulus (CR_Psych)**							
	**Total smokers**				**Male smokers**				**Total smokers**				**Male smokers**			
	**Response (CR_Peer > 0)**		**CR_Peer**		**Response (CR_Peer > 0)**		**CR_Peer**		**Response (CR_Psych > 0)**		**CR_Psych**		**Response (CR_Psych > 0)**		**CR_Psych**	
	N	%	mean	std	N	%	mean	std	N	%	mean	std	N	%	mean	std
**Total**	1,183	96.97	6.25	2.64	1,073	97.19	6.35	2.62	1,116	91.48	5.75	3.34	1,006	91.12	5.57	3.30
**Age (years)**																
18–24	120	98.36	6.36	1.93	97	97.98	6.36	1.94	119	97.54	6.99	3.05	96	96.97	6.74	3.06
25–34	198	97.06	6.23	2.52	169	97.13	6.39	2.43	193	94.61	6.89	3.37	164	94.25	6.57	3.29
35–44	282	97.24	6.01	2.57	253	97.68	6.20	2.48	268	92.41	5.88	3.22	237	91.51	5.71	3.19
45–54	236	96.33	6.45	2.78	220	96.92	6.53	2.77	222	90.61	5.65	3.34	207	91.19	5.62	3.32
55+	311	96.88	6.27	2.90	299	96.76	6.31	2.90	281	87.54	4.59	3.16	270	87.38	4.53	3.14
Missing	36				35				33				32			
**Gender**																
Woman	110	94.83	5.31	2.69					110	94.83	7.48	3.33				
Man	1,073	97.19	6.35	2.62					1,006	91.12	5.57	3.30				
**Education**																
Elementary school																
or no education	297	97.38	6.21	2.76	274	97.86	6.35	2.72	274	89.84	4.70	3.13	253	90.36	4.64	3.10
Junior high school	230	97.05	6.14	2.62	208	97.20	6.16	2.62	212	89.45	5.45	3.31	189	88.32	5.16	3.22
Senior high school	536	96.93	6.34	2.57	478	97.15	6.48	2.53	522	94.39	6.49	3.28	463	94.11	6.26	3.25
Undergraduate or graduate school	117	95.90	6.13	2.72	110	95.65	6.15	2.75	105	86.07	5.58	3.46	98	85.22	5.57	3.49
Missing	3				3				3				3			
**Employment Status**																
No	375	96.90	6.24	2.84	320	97.26	6.43	2.83	355	91.73	5.38	3.31	302	91.79	5.08	3.20
Yes	806	96.99	6.25	2.54	751	97.15	6.31	2.52	759	91.34	5.91	3.35	702	90.82	5.77	3.31
Missing	2				2				2				2			
**Social motive for the first-time smoking**																
No	928	96.97	6.22	2.63	828	97.18	6.34	2.61	882	92.16	5.95	3.34	782	91.78	5.75	3.30
Yes	255	96.96	6.37	2.69	245	97.22	6.39	2.66	234	88.97	5.02	3.28	224	88.89	4.94	3.21
**Mood motive for the first-time smoking**																
No	1,043	96.84	6.29	2.65	964	97.08	6.37	2.63	978	90.81	5.54	3.31	900	90.63	5.42	3.29
Yes	140	97.90	5.92	2.56	109	98.20	6.16	2.47	138	96.5	7.27	3.20	106	95.5	6.85	3.03
**Exposure to cigarette advertisement**																
No	548	96.82	6.15	2.79	516	96.99	6.21	2.78	509	89.93	5.27	3.28	479	90.04	5.19	3.24
Yes	635	97.09	6.34	2.50	557	97.38	6.48	2.45	607	92.81	6.16	3.35	527	92.13	5.92	3.31
**Exposure to second hand smoke in the workplace**																
No	644	96.41	6.12	2.74	598	96.45	6.18	2.75	596	89.22	5.22	3.31	550	88.71	5.07	3.28
Yes	504	97.86	6.50	2.44	474	98.14	6.55	2.42	486	94.37	6.32	3.26	455	94.2	6.20	3.22
Missing	35				1				34				1			
**Exposure to second hand smoke at home**																
No	712	97.27	6.29	2.66	679	97.42	6.34	2.65	664	90.71	5.60	3.33	632	90.67	5.53	3.30
Yes	471	96.52	6.19	2.61	394	96.81	6.37	2.56	452	92.62	5.96	3.35	374	91.89	5.63	3.29

Std: standard deviation

**Table 3a.** Regressions on smoking amount, cue-response to peer influence (CR_peer) and cue-response to psychological effect (CR_Psych) for smokers.

	**Single-equations**								
	**Smoking Amount**			**CR_peer**			**CR_Psych**		
	**Coef**	**95% CI**		**Coef**	**95% CI**		**Coef**	**95% CI**	
**Cons**	−16.37	−21.08	−11.66	6.11	5.14	7.08	7.65	6.63	8.67
**Cue-Sensitivity**									
CR_peer	1.06	0.74	1.38						
CR_Psych	0.44	0.17	0.70						
**Smoking Amount**				0.05	0.04	0.06	0.05	0.03	0.06
**Age (years) (Ref: 18–24)**									
25–34	4.64	1.72	7.56	−0.23	−0.82	0.36	−0.15	−0.86	0.55
35–44	5.67	2.86	8.49	−0.51	−1.09	0.07	−0.98	−1.66	−0.30
45–54	5.88	2.90	8.86	−0.10	−0.72	0.52	−1.02	−1.74	−0.30
55+	2.06	−1.24	5.35	−0.33	−0.99	0.34	−1.79	−2.57	−1.01
**Gender (Ref: woman)**									
man	7.56	4.88	10.23	0.50	−0.10	1.10	−1.62	−2.26	−0.98
**Education (Ref: Elementary school or no education)**									
Junior high school	−1.05	−3.6	1.50	−0.11	−0.62	0.40	0.16	−0.45	0.77
Senior high school	−3.05	−5.42	−0.67	0.15	−0.32	0.63	0.96	0.4	1.53
Undergraduate or graduate school	−6.27	−9.39	−3.14	0.09	–	0.71	0.32	−0.43	1.07
**Employment Status (Ref: No)**									
Yes	−0.7	−2.61	1.21	−0.52	−0.97	−0.07	−0.17	−0.63	0.29
**Exposure to cigarette advertisement (Ref: No)**									
Yes	−1.02	−2.65	0.60	0.37	0.04	0.69			
**Exposure to second hand smoke at home (Ref: No)**									
Yes				−0.14	−0.47	0.18			
**Exposure to second hand smoke at workplace (Ref: No)**									
Yes				0.49	0.12	0.85			
**Social motive for the first-time smoking (Ref: No)**									
Yes				0.19	−0.17	0.55			
**Mood motive for the first-time smoking (Ref: No)**									
Yes							1.25	0.68	1.82
**Smoking status (Ref: former smoking)**									
Current smoking	24.93	23.06	26.80	−1.37	−1.84	−0.91	−1.19	−1.75	−0.63
**R^2^**	**0.4631**			**0.0891**			**0.1493**		
**Hausman Test**									
χ*2* value	**1.47**			−**5.24**			**12.91**		
P-value	**1**			**–**			**0.4551**		

**Table 3b.** Regressions on smoking amount, cue-response to peer influence (CR_peer) and cue-response to psychological effect (CR_Psych) for male smokers.

	**Single-equations**								
	**Smoking Amount**			**CR_peer**			**CR_Psych**		
	**Coef**	**95% CI**		**Coef**	**95% CI**		**Coef**	**95% CI**	
**Cons**	−10.71	−15.53	−5.88	6.65	5.77	7.52	6.37	5.36	7.38
**Cue-Sensitivity**									
CR_peer	0.97	0.63	1.32						
CR_Psych	0.53	0.25	0.82						
**Smoking Amount**				0.05	0.03	0.06	0.05	0.04	0.06
**Age (years) (Ref:18–24)**									
25–34	5.25	1.97	8.53	−0.14	−0.78	0.50	−0.34	−1.12	0.44
35–44	7.22	4.09	10.35	−0.35	−0.98	0.27	−1.12	−1.86	−0.38
45–54	7.17	3.89	10.44	−0.01	−0.66	0.64	−1.09	−1.87	−0.32
55+	3.42	−0.15	6.99	−0.27	−0.97	0.43	−2.03	−2.86	−1.20
**Education (Ref: Elementary school or no education)**									
Junior high school	−0.76	−3.48	1.96	−0.23	−0.76	0.30	−0.13	−0.77	0.51
Senior high school	−3.07	−5.60	−0.55	0.14	−0.35	0.63	0.80	0.21	1.39
Undergraduate or graduate school	−6.35	−9.64	−3.06	−0.03	−0.67	0.62	0.30	−0.47	1.07
**Employment Status (Ref: No)**									
Yes	−1.15	−3.26	0.96	−0.55	−1.02	−0.08	−0.18	−0.68	0.31
**Exposure to cigarette advertisement (Ref: No)**									
Yes	−0.83	−2.56	0.89	0.37	0.04	0.70			
**Exposure to second hand smoke at home (Ref: No)**									
Yes				−0.09	−0.43	0.24			
**Exposure to second hand smoke at workplace (Ref: No)**									
Yes				0.50	0.12	0.88			
**Social motive for the first-time smoking (Ref: No)**									
Yes				0.12	−0.25	0.49			
**Mood motive for the first-time smoking (Ref: No)**									
Yes							1.18	0.54	1.81
**Smoking status (Ref: former smoking)**									
Current smoking	26.14	24.13	28.15	−1.38	−1.87	−0.9	−1.3	−1.89	−0.7
**R^2^**	**0.4658**			**0.0763**			**0.1252**		
**Hausman Test**									
χ^2^ value	**−940.21**			**−848.21**			**−938.04**		
P-value		**–**			**–**			**–**	
